# Visible‐Light‐Driven CO_2_ Reduction by Mesoporous Carbon Nitride Modified with Polymeric Cobalt Phthalocyanine

**DOI:** 10.1002/anie.201907082

**Published:** 2019-07-30

**Authors:** Souvik Roy, Erwin Reisner

**Affiliations:** ^1^ Christian Doppler Laboratory for Sustainable Syngas Chemistry Department of Chemistry University of Cambridge Cambridge CB2 1EW UK

**Keywords:** carbon nitride, catalyst immobilization, CO_2_ reduction, cobalt phthalocyanine, photocatalysis

## Abstract

The integration of molecular catalysts with low‐cost, solid light absorbers presents a promising strategy to construct catalysts for the generation of solar fuels. Here, we report a photocatalyst for CO_2_ reduction that consists of a polymeric cobalt phthalocyanine catalyst (CoPPc) coupled with mesoporous carbon nitride (mpg‐CN_*x*_) as the photosensitizer. This precious‐metal‐free hybrid catalyst selectively converts CO_2_ to CO in organic solvents under UV/Vis light (AM 1.5G, 100 mW cm^−2^, λ>300 nm) with a cobalt‐based turnover number of 90 for CO after 60 h. Notably, the photocatalyst retains 60 % CO evolution activity under visible light irradiation (λ>400 nm) and displays moderate water tolerance. The in situ polymerization of the phthalocyanine allows control of catalyst loading and is key for achieving photocatalytic CO_2_ conversion.

Photocatalytic reduction of CO_2_ to produce storable fuels offers an attractive path to capture and utilize the greenhouse gas CO_2_ and ultimately implement a carbon‐neutral energy cycle. The development of efficient, sustainable, and economically viable catalysts and light‐absorbers lies at the nexus of solar‐fuel research on CO_2_ utilization. Hybrid photosynthetic systems with molecular catalysts immobilized on solid supports (light‐absorbing semiconductors or dye‐sensitized semiconductors) have recently emerged as a promising approach for suspension‐based photoreactor applications, because they combine the selectivity of molecules with the durability of heterogeneous materials.^1^ While many earth abundant metal based molecular complexes have been reported for CO_2_ reduction in homogeneous solution, there are relatively few examples of heterogenization of these catalysts on solid light‐absorbers.^2^ The development of new robust catalyst–photosensitizer interfaces remains a challenge that offers the key for improved photocatalytic activity of colloidal material–molecule hybrid systems.

Graphitic carbon nitride (g‐CN_*x*_) has recently emerged as a promising semiconductor for photocatalytic applications,^3^ including water splitting^4^ and CO_2_ reduction,^5^ because of its nontoxicity, facile synthesis, capability to absorb UV as well as visible light, and durability under photochemical conditions. A relatively narrow band gap and sufficiently negative conduction band energy minimum (−1.10 V vs. NHE at pH 6.6)4b, 6 allow g‐CN_*x*_ to harvest UV/Vis light and subsequently reduce a surface‐bound molecular catalyst via photoinduced electron transfer. In CN_*x*_‐based photocatalytic systems for CO_2_ reduction, different types of co‐catalysts have been used, including weakly anchoring phosphonic acid functionalized Ru complexes or Ru‐Re dyads,^6^, ^7^ molecular cobalt and iron complexes in solution,^8^ metalloporphyrins covalently grafted on CN_*x*_,^9^ single‐atom cobalt sites incorporated in the material,^10^ and sodium niobite nanowires.^11^ Despite encouraging reports with CN_*x*_–porphyrin hybrid catalysts,^9^, ^12^ a CN_*x*_/molecular catalyst system that consists of only earth‐abundant elements and is entirely heterogeneous, durable, efficient, and selective for CO production remains an elusive target. Cobalt phthalocyanine is a known electrocatalyst for CO_2_ reduction^13^ but has rarely been explored in photocatalysis.^14^

Herein, we report photocatalytic reduction of CO_2_ to CO by a robust organic–inorganic hybrid material in which mesoporous carbon nitride (mpg‐CN_*x*_) harvests solar energy and activates a surface‐deposited polymeric cobalt phthalocyanine (CoPPc; PPc denotes *p*olymeric *p*hthalo*c*yanine) catalyst toward CO_2_ reduction (Figure 1). CoPPc is deposited on mpg‐CN_*x*_ via an in situ polymerization method, which represents an effective strategy for catalyst immobilization. This study demonstrates that the polymer–CN_*x*_ interface plays a key role in catalysis by enabling the transfer of photoexcited electrons from CN_*x*_ to the attached CoPPc catalyst.

**Figure 1:**
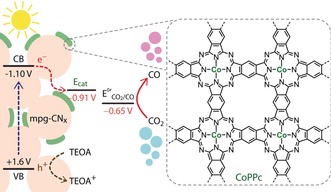
Schematic illustration of light‐driven CO_2_ reduction catalyzed by mpg‐CN_*x*_|CoPPc hybrid (potentials reported against NHE; VB and CB denote valence and conduction band, respectively).

Mpg‐CN_*x*_ was prepared by heating cyanamide in air using colloidal silica as a hard template, which was subsequently etched with aqueous ammonium bifluoride.^15^ The mpg‐CN_*x*_|CoPPc hybrid was synthesized by microwave‐assisted polymerization of 1,2,4,5‐tetracyanobenzene (TCNB) with Co^2+^ ions in the presence of mpg‐CN_*x*_ dispersed in 1‐pentanol.^16^ Formation of the polymeric catalyst was observed by a color change from pale orange to green. A weak π–π stacking interaction between the polymeric phthalocyanine sheet and tri‐s‐triazine units of mpg‐CN_*x*_ may contribute toward the facile charge transport through the interface.^17^ The cobalt content in the hybrids (mpg‐CN_*x*_|CoPPc_*a*_; *a*=μmol Co g^−1^) was modulated by controlling the amount of TCNB during the synthesis. Catalyst loadings, determined by inductively coupled plasma optical emission spectrometry (ICP‐OES), were in the range of 4.1–107 μmol Co g^−1^ with higher Co content causing a visible intensification of green color of the solid (Figure S1).

Successful formation of CoPPc on mpg‐CN_*x*_ was confirmed by diffuse reflectance UV/Vis spectroscopy (DRS), attenuated total reflectance infrared (ATR‐IR), Raman, and X‐ray photoelectron spectroscopy (XPS). DRS of mpg‐CN_*x*_|CoPPc shows the characteristic (S_0_→S_1_) Q band of CoPPc at 700 nm, which is red‐shifted compared to the monomeric cobalt phthalocyanine (CoPc) (670 nm in DMF), consistent with its polymeric structure (Figure 2 A).16a, 18 Figure 2 B shows that the Raman spectrum of mpg‐CN_*x*_ is featureless, whereas that of the hybrid materials displays bands originating from CoPPc.^19^ The ATR‐IR spectrum of mpg‐CN_*x*_|CoPPc is dominated by mpg‐CN_*x*_ peaks, which mask the weaker CoPPc stretches (Figure S2).

**Figure 2:**
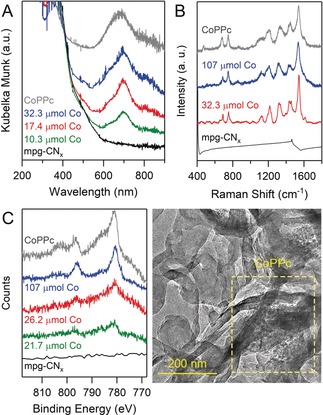
A) UV/Vis DRS, B) Raman spectra, and C) Co 2p region of the XPS of bare mpg‐CN_*x*_, CoPPc, and mpg‐CN_*x*_|CoPPc with varying catalyst loading. D) TEM image of mpg‐CN_*x*_|CoPPc_17.4_.

The introduction of CoPPc leads to a lower Brunauer–Emmett–Teller (BET) surface area of the hybrid material (104 m^2^ g^−1^ for mpg‐CN_*x*_|CoPPc_21.7_ vs. 134 m^2^ g^−1^ for bare mpg‐CN_*x*_, Figure S3), suggesting that the catalyst deposition occurs not only on the surface, but also inside the mesoporous structure. However, the integration of CoPPc does not affect the periodic stacking of the lamellar structure of CN_*x*_, as demonstrated by powder X‐ray diffraction (Figure S4). XPS spectra of pure CoPPc polymer and mpg‐CN_*x*_|CoPPc at three different catalyst loadings are shown in Figure 2 C and Figure S5. The Co 2p region of all samples consists of peaks at 796.2 and 780.8 eV associated with Co 2p_1/2_ and Co 2p_3/2_ transitions, respectively. The satellite features at ≈802 and ≈786 eV, which are characteristic of Co^II^ paramagnetic species, are clearly discernible for CoPPc and mpg‐CN_*x*_|CoPPc_107_, but are less pronounced for lower cobalt contents. The C 1s XPS spectra of mpg‐CN_*x*_|CoPPc feature a more intense peak at 284.8 eV, which can be attributed to the C(sp^2^)–C(sp^2^) bonds of CoPPc (Figure S6). The intensity of this peak increases with higher catalyst loading.

Transmission electron microscopy (TEM, Figure 2 D, S7), scanning electron microscopy (SEM, Figure S8) and energy‐dispersive X‐ray (EDX) analysis (Figures S7–S9) of mpg‐CN_*x*_|CoPPc confirm a uniform distribution of Co throughout the material (particle size ca. 200–500 nm from SEM). TEM images of the hybrid reveal CoPPc material as a network, interweaved with CN_*x*_ domain (Figure 2 D and Figure S7).

The overpotential required for CO_2_ reduction by CoPPc was estimated by cyclic voltammetric analysis of CoPPc/carbon nanotube (CNT) composite electrodes.13c The cyclic voltammogram of CoPPc|CNT in a mixture of acetonitrile (MeCN) and triethanolamine (TEOA) (4:1 v/v) displays a large catalytic wave under CO_2_ with an onset at −0.91 V vs. NHE (Figure S11), which suggests that CO_2_ reduction by CoPPc occurs at a more positive potential compared to the conduction band of CN_*x*_.

The photocatalytic activity of the mpg‐CN_*x*_|CoPPc hybrid was studied in CO_2_‐saturated MeCN under UV‐filtered simulated solar light irradiation (100 mW cm^−2^, AM 1.5G, *λ*>400 nm) with TEOA as a sacrificial electron donor. While bare mpg‐CN_*x*_ generates only trace amounts of H_2_ and CO (headspace analysis by gas chromatography), the CoPPc‐modified material (mpg‐CN_*x*_|CoPPc) exhibits considerably higher activity toward CO_2_ reduction to CO (red trace, Figure 3 A). Mechanically mixed pure CoPPc and mpg‐CN_*x*_ is inactive toward CO_2_ reduction (blue trace, Figure 3 A), highlighting the importance of the in situ polymerization. Only trace amounts of formate (<1 μmol g^−1^) were detected in all systems by ion chromatography.

**Figure 3:**
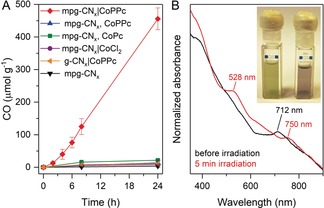
A) CO generation by mpg‐CN_*x*_|CoPPc_12_ (2 mg) in CO_2_‐saturated 4:1 MeCN/TEOA under visible light (100 mW cm^−2^, AM 1.5G, *λ*>400 nm) (red trace). Controls: green: mpg‐CN_*x*_ with commercial CoPc (0.15 nmol mg^−1^) in solution; blue: mechanically mixed mpg‐CN_*x*_ and CoPPc (0.6 wt %); purple: mpg‐CN_*x*_|CoCl_2_ synthesized by microwave heating; orange: non‐mesoporous CN_*x*_ (g‐CN_*x*_) with CoPPc; black: bare mpg‐CN_*x*_. B) UV/Vis spectra of a mpg‐CN_*x*_|CoPPc_107_ suspension in MeCN/TEOA (0.11 mg mL^−1^) before and after illumination (*λ*>400 nm, 5 min) under CO_2_. Photographs of the suspension before and after illumination are shown in the inset.

This hypothesis is further supported by the low CO evolution activity of a suspension of mpg‐CN_*x*_ with monomeric CoPc in solution (green trace). CO was not observed in control experiments without mpg‐CN_*x*_, TEOA, CO_2_, or light. Isotope labeling studies with ^13^CO_2_ using mass spectrometry and infrared spectroscopy confirmed that CO was produced from CO_2_ (Figures S12 and S13). Irradiation of mpg‐CN_*x*_|CoPPc under visible light equipped with a long‐pass filter (λ>455 nm) produced very little CO, which is consistent with mpg‐CN_*x*_ acting as the light absorber. To confirm that the cobalt phthalocyanine units in the polymer are the active catalyst and not single‐site cobalt ions coordinated to the tri‐s‐triazine moieties of CN_*x*_,^10^ mpg‐CN_*x*_|CoCl_2_ was synthesized by heating CoCl_2_ and mpg‐CN_*x*_ in the absence of TCNB under identical reaction conditions (weight ratio 1:120; equivalent to CoCl_2_ used for mpg‐CN_*x*_|CoPPc_26.2_ synthesis). This material displays minimal activity toward CO_2_ reduction (purple trace). A non‐mesoporous carbon‐nitride‐based hybrid (g‐CN_*x*_|CoPPc) is also inactive under identical condition (orange trace).

The hybrid catalysts change color from pale green to purple during photocatalysis, indicating photoreduction of the Co centers of CoPPc (Figure 3 B, inset). UV/Vis absorption spectra of an acetonitrile suspension (20 % TEOA, v/v) of mpg‐CN_*x*_|CoPPc_107_ display a red‐shift of the Q‐band of CoPPc from 712 to 750 nm and appearance of a new charge transfer band at 528 nm, upon visible light irradiation under CO_2_ (Figure 3 B, red trace). The spectral change suggests formation of a reduced CoPPc species, which was corroborated by spectroelectrochemical analysis of a CoPPc thin film deposited on conductive FTO (fluorine‐doped tin oxide)‐coated glass electrode (CoPPc|FTO, Figure S14). An electrochemically reduced CoPPc film (−1.0 V vs. NHE) exhibits two absorption bands at 529 and 735 nm, which is consistent with the spectrum of Co^I^PPc.^20^ This indicates transfer of the photoexcited electron from the conduction band of CN_*x*_ to CoPPc to yield Co^I^ centers that subsequently bind CO_2_ and convert it to CO through a second electron transfer from (mpg‐CN_*x*_)* or (mpg‐CN_*x*_)^−^ (Figure S15).13c, 21

Photocatalytic activity of mpg‐CN_*x*_|CoPPc is largely dependent on the catalyst loading as illustrated in Figure 4 A. The amount of CO generated increased linearly with cobalt loading until ≈12 μmol Co g^−1^. Further increase in cobalt content (>20 μmol g^−1^) resulted in a decrease in activity and only a trace amount of CO was detected for the highest loading sample (107 μmol Co g^−1^). At high cobalt concentrations, the carbon nitride surface is completely sheathed by the CoPPc layer, which blocks the incoming light and reduces the accessibility of the mpg‐CN_*x*_ surface to TEOA, thereby hindering photocatalysis. The amount of CO evolved vs. Co loading profile fits well with the selectivity toward CO exhibited by mpg‐CN_*x*_|CoPPc (Figure 4 A, Table S2). Long‐term photocatalysis experiments show that the catalyst remains active for 4 days and only displays a marginal decrease of CO selectivity, highlighting the stability and robustness of the photocatalyst assembly (Table S2 and Figure S16).

**Figure 4:**
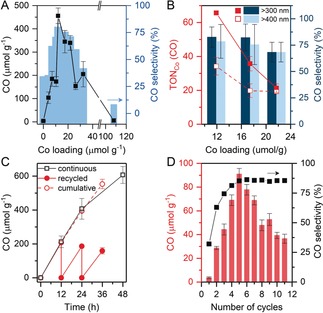
A) Dependence of the amount of CO evolved (black trace) and selectivity towards CO (blue bars) on Co loading (CO_2_‐saturated MeCN/TEOA, 24 h, 100 mW cm^−2^, AM 1.5G, *λ*>400 nm). B) Co‐based turnover numbers (TON_Co_) for CO evolution after 48 h under visible (*λ*>400 nm, dashed red trace) and UV/Vis (*λ*>300 nm, solid red trace) light; bar plot shows the CO selectivity. C,D) Catalyst recycling experiments performed under visible light. C) The solid and dashed red traces display the CO evolved during three cycles and the cumulative CO, respectively, with mpg‐CN_*x*_|CoPPc_11.9_ (≈2 mg). The black trace shows the CO accumulated during continuous irradiation. D) CO evolved during ten 4 h recycling runs with mpg‐CN_*x*_|CoPPc_27_ (≈8 mg) is plotted as bars and the black trace shows the selectivity.

Under full solar spectrum irradiation (*λ*>300 nm), mpg‐CN_*x*_|CoPPc_11.9_ generated 1000 μmol CO g^−1^ after 48 h with 85 % selectivity (TON_Co_=84), which corresponds to a 65 % increase in activity compared to that under visible light alone (607 μmol CO g^−1^ after 48 h, TON_Co_=51) (Figure 4 B, Figure S17, and Table S3). Similarly, mpg‐CN_*x*_|CoPPc_17.4_ exhibited a 45 % increase in activity under UV/Vis irradiation and a small improvement in selectivity toward CO. This observation is consistent with the high UV absorbance (<400 nm) of mpg‐CN_*x*_. However, this enhancement becomes less pronounced at higher Co loading as demonstrated by the similar TON_Co_ values observed for mpg‐CN_*x*_|CoPPc_21.7_ under visible and UV/Vis irradiation (Figure 4 B, red traces). The external quantum efficiency (EQE) for CO formation by mpg‐CN_*x*_|CoPPc_11.9_ was calculated to be 0.11 % and 0.03 % at *λ_ex_*=360 and 400 nm, respectively (see Table S4 and Figure S18 for more information).

The amount of CO evolved in three 12 h recycling tests displays excellent agreement with that produced during continuous irradiation under visible light, consistent with heterogenous catalysis (Figure 4 C). When the runtime for each cycle was shortened to 4 h, the catalyst displayed a significant induction period and the CO evolution peaked during the 5^th^ cycle with subsequent gradual loss of activity. The use of a larger amount of catalyst is likely responsible for the delay as light scattering in concentrated suspension becomes a limiting factor. However, the catalyst retains its excellent selectivity up to the 11^th^ cycle (44 h). In a long‐term experiment, the catalyst was recycled after 26 h visible light photocatalysis and it retained 84 % activity in the second run for 38 h (Figure S19).

Monitoring the cobalt content of the mpg‐CN_*x*_|CoPPc_17.4_ under visible light photocatalysis reveals ≈20 % loss of cobalt over the initial 4 h (Figure S20). However, very little Co subsequently leached from the photocatalyst between 4 and 48 h. XPS analysis of the catalyst after 24 h photocatalysis confirms that Co remains on the mpg‐CN_*x*_ surface (Figure S21).

Addition of water to the reaction medium affects the performance of the photocatalyst. In comparison to the photocatalytic experiments in which CO is produced by mpg‐CN_*x*_|CoPPc_21.7_ under visible light in MeCN, the activity drops to 41 % and 26 % in the presence of 10 % and 20 % water, respectively (Figures S22 and 23). The reduced activity in water is likely caused by the phase separation of MeCN/H_2_O/TEOA mixture during photocatalysis, with mpg‐CN_*x*_|CoPPc being partitioned into the bottom aqueous layer (Figure S22 C).^22^ When dimethylacetamide (DMA) was used as the solvent,^23^ the reaction mixture remained monophasic and the photocatalyst exhibited markedly improved water tolerance (Figures S24 and 25). Compared to the experiments in DMA, mpg‐CN_*x*_|CoPPc_11.9_ retains 90 % and 78 % CO evolution activity in the presence of 10 % and 20 % water, respectively. Under fully aqueous conditions, the photocatalyst produced 62 μmol CO g^−1^, corresponding to ≈5.1 turnovers per Co. Water‐tolerance is an important feature as it may enable the photoreduction of CO_2_ using water as an electron donor in the future.^24^

Previously reported CN_*x*_‐based photocatalysts for CO_2_ reduction commonly employed either molecular co‐catalysts that remained in the solution phase or phosphonic acid functionalized Ru complexes and Ru/Re dyads that interact weakly with the CN_*x*_ via its surface ‐NH_2_ groups.7b, 8a, 23 Only a few examples of hybrid materials with immobilized molecular catalysts have been reported, including a Co‐porphyrin covalently attached to CN_*x*_,^9^ and a mechanically mixed Fe‐porphyrin/CN_*x*_ hybrid.^12^ In the former case, the TON_Co_ and selectivity were reported to be <1 and ≈80 % under 80 kPa CO_2_, respectively, whereas in the latter case, high CO selectivity (98 %) was observed with a TON of 5.7. In comparison, the mpg‐CN_*x*_|CoPPc system described here performs well over longer time periods with 90 turnovers after 60 h and displays moderate tolerance toward water. However, this rate is still significantly slower than electrocatalytic CO evolution rates displayed by CoPPc|CNT composite electrodes (TON 11 240, 24 h electrolysis),13c suggesting that photocatalysis is likely limited by the rate of transfer of electron from mpg‐CN_*x*_ to the catalyst and not the inherent CO_2_ reduction capability of CoPPc. This is supported by the linear decrease of photocatalytic activity of mpg‐CN_*x*_|CoPPc with light intensity, while the selectivity remained unaffected (Figure S26). Notably, the mesoporosity of CN_*x*_ plays a key role in catalysis by facilitating electron–hole separation through shortening of the migration distance.^25^

In summary, we have interfaced 2D cobalt phthalocyanine sheets with mesoporous carbon nitride via an in situ polymerization technique to fabricate a hybrid catalyst for use in selective CO_2_ reduction under visible light irradiation. Photocatalysis and spectroscopic studies demonstrate that molecular cobalt phthalocyanine units act as the catalytic centers and that the catalysis is enabled by the immobilization of the polymer in the porous carbon nitride. This work provides a rare example of an effective and robust heterogenous CO_2_ reduction photocatalyst featuring inexpensive, earth‐abundant components, and provides a versatile platform for catalyst immobilization on heterogeneous light absorbers.

## Conflict of interest

The authors declare no conflict of interest.

## Supporting information

As a service to our authors and readers, this journal provides supporting information supplied by the authors. Such materials are peer reviewed and may be re‐organized for online delivery, but are not copy‐edited or typeset. Technical support issues arising from supporting information (other than missing files) should be addressed to the authors.

Supplementary

## References

[1a] T. Morikawa , S. Sato , K. Sekizawa , T. Arai , T. M. Suzuki , ChemSusChem 2019, 12, 1807–1824; 30963707 10.1002/cssc.201900441

[1b] J. Willkomm , K. L. Orchard , A. Reynal , E. Pastor , J. R. Durrant , E. Reisner , Chem. Soc. Rev. 2016, 45, 9–23; 26584204 10.1039/c5cs00733j

[1c] N. Elgrishi , M. B. Chambers , X. Wang , M. Fontecave , Chem. Soc. Rev. 2017, 46, 761–796; 28084485 10.1039/c5cs00391a

[1d] C. D. Windle , E. Reisner , Chimia 2015, 69, 435–441. 10.2533/chimia.2015.435 28482976

[2a] K. E. Dalle , J. Warnan , J. J. Leung , B. Reuillard , I. S. Karmel , E. Reisner , Chem. Rev. 2019, 119, 2752–2875; 30767519 10.1021/acs.chemrev.8b00392 PMC6396143

[2b] M. F. Kuehnel , K. L. Orchard , K. E. Dalle , E. Reisner , J. Am. Chem. Soc. 2017, 139, 7217–7223; 28467076 10.1021/jacs.7b00369

[2c] S. Lian , M. S. Kodaimati , E. A. Weiss , ACS Nano 2018, 12, 568–575; 29298382 10.1021/acsnano.7b07377

[2d] Q.-Q. Bi , J.-W. Wang , J.-X. Lv , J. Wang , W. Zhang , T.-B. Lu , ACS Catal. 2018, 8, 11815–11821;

[2e] K. Li , B. Peng , T. Peng , ACS Catal. 2016, 6, 7485–7527.

[3a] Y. Wang , X. Wang , M. Antonietti , Angew. Chem. Int. Ed. 2012, 51, 68–89; 10.1002/anie.201101182 22109976

[3b] W.-J. Ong , L.-L. Tan , Y. H. Ng , S.-T. Yong , S.-P. Chai , Chem. Rev. 2016, 116, 7159–7329. 27199146 10.1021/acs.chemrev.6b00075

[4a] J. Liu , Y. Liu , N. Liu , Y. Han , X. Zhang , H. Huang , Y. Lifshitz , S.-T. Lee , J. Zhong , Z. Kang , Science 2015, 347, 970–974; 25722405 10.1126/science.aaa3145

[4b] X. Wang , K. Maeda , A. Thomas , K. Takanabe , G. Xin , J. M. Carlsson , K. Domen , M. Antonietti , Nat. Mater. 2009, 8, 76–80. 18997776 10.1038/nmat2317

[5a] Y. Fang , X. Wang , Chem. Commun. 2018, 54, 5674–5687; 10.1039/c8cc02046a 29745388

[5b] K. Li , B. Peng , J. Jin , L. Zan , T. Peng , Appl. Catal. B 2017, 203, 910–916.

[6] K. Maeda , K. Sekizawa , O. Ishitani , Chem. Commun. 2013, 49, 10127–10129. 10.1039/c3cc45532g 24048317

[7a] R. Kuriki , M. Yamamoto , K. Higuchi , Y. Yamamoto , M. Akatsuka , D. Lu , S. Yagi , T. Yoshida , O. Ishitani , K. Maeda , Angew. Chem. Int. Ed. 2017, 56, 4867–4871; 10.1002/anie.201701627 28387039

[7b] R. Kuriki , K. Sekizawa , O. Ishitani , K. Maeda , Angew. Chem. Int. Ed. 2015, 54, 2406–2409; 10.1002/anie.201411170 25565575

[7c] C. Tsounis , R. Kuriki , K. Shibata , J. J. M. Vequizo , D. Lu , A. Yamakata , O. Ishitani , R. Amal , K. Maeda , ACS Sustainable Chem. Eng. 2018, 6, 15333–15340.

[8a] J. Lin , Z. Pan , X. Wang , ACS Sustainable Chem. Eng. 2014, 2, 353–358;

[8b] C. Cometto , R. Kuriki , L. Chen , K. Maeda , T.-C. Lau , O. Ishitani , M. Robert , J. Am. Chem. Soc. 2018, 140, 7437–7440. 29888920 10.1021/jacs.8b04007

[9] G. Zhao , H. Pang , G. Liu , P. Li , H. Liu , H. Zhang , L. Shi , J. Ye , Appl. Catal. B 2017, 200, 141–149.

[10] P. Huang , J. Huang , S. A. Pantovich , A. D. Carl , T. G. Fenton , C. A. Caputo , R. L. Grimm , A. I. Frenkel , G. Li , J. Am. Chem. Soc. 2018, 140, 16042–16047. 30415539 10.1021/jacs.8b10380

[11] H. Shi , G. Chen , C. Zhang , Z. Zou , ACS Catal. 2014, 4, 3637–3643.

[12] L. Lin , C. Hou , X. Zhang , Y. Wang , Y. Chen , T. He , Appl. Catal. B 2018, 221, 312–319.

[13a] C. M. Lieber , N. S. Lewis , J. Am. Chem. Soc. 1984, 106, 5033–5034;

[13b] N. Morlanés , K. Takanabe , V. Rodionov , ACS Catal. 2016, 6, 3092–3095;

[13c] N. Han , Y. Wang , L. Ma , J. Wen , J. Li , H. Zheng , K. Nie , X. Wang , F. Zhao , Y. Li , J. Fan , J. Zhong , T. Wu , D. J. Miller , J. Lu , S.-T. Lee , Y. Li , Chem 2017, 3, 652–664.

[14] J. Grodkowski , T. Dhanasekaran , P. Neta , P. Hambright , B. S. Brunschwig , K. Shinozaki , E. Fujita , J. Phys. Chem. A 2000, 104, 11332–11339.

[15] F. Goettmann , A. Fischer , M. Antonietti , A. Thomas , Angew. Chem. Int. Ed. 2006, 45, 4467–4471; 10.1002/anie.200600412 16770823

[16a] D. Wöhrle , E. Preußner , Makromol. Chem. 1985, 186, 2189–2207;

[16b] M. N. Kopylovich , V. Y. Kukushkin , M. Haukka , K. V. Luzyanin , A. J. L. Pombeiro , J. Am. Chem. Soc. 2004, 126, 15040–15041. 15547996 10.1021/ja046759i

[17] X.-W. Song , H.-M. Wen , C.-B. Ma , H.-H. Cui , H. Chen , C.-N. Chen , RSC Adv. 2014, 4, 18853–18861.

[18] B. Ortiz , S. M. Park , N. Doddapaneni , J. Electrochem. Soc. 1996, 143, 1800–1805.

[19] M. Szybowicz , W. Bała , K. Fabisiak , K. Paprocki , M. Drozdowski , Cryst. Res. Technol. 2010, 45, 1265–1271.

[20] W. A. Nevin , M. R. Hempstead , W. Liu , C. C. Leznoff , A. B. P. Lever , Inorg. Chem. 1987, 26, 570–577.

[21] M. Zhu , R. Ye , K. Jin , N. Lazouski , K. Manthiram , ACS Energy Lett. 2018, 3, 1381–1386.

[22] J. Lin , R. Liao , J. Xu , RSC Adv. 2018, 8, 3798–3802. 10.1039/c7ra12801k PMC9077867 35542908

[23] R. Kuriki , H. Matsunaga , T. Nakashima , K. Wada , A. Yamakata , O. Ishitani , K. Maeda , J. Am. Chem. Soc. 2016, 138, 5159–5170. 27027822 10.1021/jacs.6b01997

[24] S. Sato , T. Arai , T. Morikawa , Inorg. Chem. 2015, 54, 5105–5113. 25679545 10.1021/ic502766g

[25] K. Maeda , R. Kuriki , M. Zhang , X. Wang , O. Ishitani , J. Mater. Chem. A 2014, 2, 15146–15151.

