# Supplementary material for: Visible‐Light‐Driven CO2 Reduction by Mesoporous Carbon Nitride Modified with Polymeric Cobalt Phthalocyanine
Source: Angew Chem Int Ed Engl. 2019 Jul 30;58(35):12180–4. doi: 10.1002/anie.201907082 (PMC6771752; doi:10.1002/anie.201907082)
Supplement: Supplementary file 1 — Supplementary [file ANIE-58-12180-s001.pdf]

Angewandte Chemie
Eine Zeitschrift der Gesellschaft Deutscher Chemiker

Supporting Information

Visible-Light-Driven CO_2 Reduction by Mesoporous Carbon Nitride Modified with Polymeric Cobalt Phthalocyanine

*Souvik Roy and Erwin Reisner**

anie_201907082_sm_miscellaneous_information.pdf

Experimental Procedures

Materials. All chemicals were purchased from commercial suppliers and used without further purification. mpg-CN_x and g-CN_x were synthesized from cyanamide and characterized by UV-vis and infrared spectroscopy, X-ray diffraction, and Brunauer-Emmett-Teller (BET) measurement as previously described.[1]

Synthesis of mpg-CN_x|CoPPc. To a dispersion of mpg-CN_x (115 mg) in 1-pentanol (10 mL) (prepared by 15 min ultra-sonication), 1,2,4,5-tetracyanobenzene (TCNB, 2.5 mg, 0.014 mmol, 1 equiv.), anhydrous $CoCl_2$ (1.1 mg, 0.0085 mmol, 0.6 equiv.) and 1,8-diazabicyclo[5.4.0] undec-7-ene (DBU) (2.3 mg, 0.015 mmol, 1 equiv.) was added under inert atmosphere, and the dispersion was heated under microwave radiation at 180 °C for 2.5 h. The color of the suspension changed from yellow/orange to pale green (dark yellow for low cobalt loading). The final product was collected by filtration and sequentially washed with ethanol, hot water, boiling ethanol, boiling methanol, boiling acetone, and dried at 80 °C under vacuum. A cobalt loading of 0.1546 wt.% (26.2 μmol Co g^{-1}) was determined by ICP-OES. Hybrids with different CoPPc loadings from 0.0241 wt.% (4.1 μmol Co g^{-1}) to 0.6333 wt.% (107.3 μmol Co g^{-1}) were similarly prepared by adjusting the amounts of mpg-CN_x and the precursors (Table S5).

Hybrid material with non-mesoporous carbon nitride (g-CN_x) was synthesized under identical condition using 200 mg g-CN_x, 3.8 mg 1,2,4,5-tetracyanobenzene, 1.5 mg anhydrous $CoCl_2$, and 3.1 mg DBU in 9 mL 1-penatanol. The cobalt content was determined to be 0.2 wt.% by ICP-OES.

Pure CoPPc was prepared by identical method in the absence of carbon nitride. The Co content in CoPPc was determined to be 7.47 wt.% by ICP-OES. Mpg-CN_x and CoPPc (0.6 wt.%) were mechanically ground together to prepare the composite catalyst (mpg-CN_x, CoPPc) that was tested for photocatalytic activity (Figure 3A, blue trace, main manuscript).

Preparation of CoPPc|FTO. A clean FTO-coated glass slide was placed inside a glass vial containing TCNB (12 mg), anhydrous $CoCl_2$ (5.4 mg), DBU (11.6 mg) in 2 mL 1-pentanol. The solution was heated at 160 °C under N_2 for 20 h. The CoPPc|FTO electrode was removed and briefly sonicated in acetone. Prior to spectroelectrochemical measurements, the film attached to the non-conductive surface of the FTO slide was carefully removed by wiping the surface with a wet Q-tip (1 M HCl) and subsequent wash with water and acetone.

Synthesis of mpg-CN_x|$CoCl_2$. mpg-CN_x (120 mg), anhydrous $CoCl_2$ (1 mg), and DBU (2.5 mg) were dispersed in 1-pentanol (8 mL) by ultra-sonication and heated under microwave at 180°C for 2.5 h. The resulting yellow solid was collected by filtration and sequentially washed with ethanol, hot water, boiling ethanol, boiling methanol, boiling acetone, and dried at 80°C under vacuum. The cobalt content was determined to be 0.036 wt.% by ICP-OES.

Synthesis of CoPPc|CNT (1:1 wt. ratio). The composite was prepared by literature method.[2]

Characterization and Physical Measurements. Fourier transform infrared spectra (FT-IR) were recorded on a Thermo Scientific Nicolet iS50 FT-IR spectrometer operating in an ATR mode. UV−visible spectroscopy was carried out on a Varian Cary 50 UV−vis spectrophotometer using quartz cuvettes with 1 cm path length. Scanning electron microscopy (SEM) images were collected on MIRA3 FEG-SEM (TESCAN) at an accelerating voltage of 5 kV. Energy-dispersive X-ray (EDX) spectra were recorded using an Oxford Instruments X-Max silicon drift detector. Raman spectra were recorded on a HORIBA LabRAM HR Evolution system with an incident laser of 633 nm. X-ray photoelectron spectroscopy (XPS) was performed on a Thermo Fisher Scientific K-alpha$^+$ spectrometer. Samples were prepared by dropcasting 5 mg mL^{-1} suspension of the catalysts on clean FTO slides. Samples were analyzed using a micro-focused monochromatic Al x-ray source over an area of approximately 200 microns. Data were recorded at pass energies of 150 eV for survey scans and 40 eV for a high resolution scan with 1 eV and 0.1 eV step sizes, respectively. Charge neutralization of the sample was achieved using a combination of both low energy electrons and argon ions. Data analysis was performed in CasaXPS using a Shirley type background and Scofield cross sections, with an energy dependence of –0.6. Brunauer-Emmett-Teller (BET) measurements were performed on a 3Flex Surface Characterization Analyzer (Micromeritics) at 77 K. Prior to measurements, the

samples were degassed under vacuum for 6 h at 120 °C. Powder XRD pattern of mpg-CN_x and hybrid materials were collected on a Panalytical Empyrean diffractometer (Cu radiation). Inductively-coupled plasma-optical emission spectroscopy (ICP-OES) was carried-out at the Department of Geography, University of Cambridge with a Perkin-Elmer ICP-OES chemical analyzer. mpg-CN_x|CoPPc samples (1–5 mg) were digested in 2:1 mixture of H_2O_2 (30% v/v) solution and conc. H_2SO_4 and diluted by ultra-pure water to (0–10) ppm analyte. Cyclic voltammetry was performed on a PalmSens EmStat potentiostat using a conventional three electrode set up with a Pt-wire counter electrode, and a Ag/AgCl/KCl (sat'd) reference electrode. A 0.1 M solution of NBu_4^+ PF_6^- in MeCN/TEOA was used as supporting electrolyte. The working electrode was prepared by dropcasting 5 µL catalyst ink (1 mg CoPPc|CNT dispersed in a mixture of 0.5 mL 2-propanol and 5 µL 10 wt.% nafion solution) on a glassy carbon electrode and drying under air. The reference electrode was calibrated using ferrocene as internal standard and the potential was converted from Ag/AgCl/KCl (sat'd) to NHE (noramal hydrogen electrode) by adding 0.197 V. The spectroelectrochemical measurement was conducted by holding CoPPc|FTO electrode at –1.0 V vs NHE (0.1 M NBu_4^+ PF_6^- in MeCN as supporting electrolyte); the UV-vis spectra were recorded in transmission mode for 6 min at ~1 min intervals.

Photocatalytic Experiments. In photocatalytic CO_2 reduction experiments, a specific amount of catalyst was dispersed in a 3.0 mL 4:1 MeCN/TEOA (v/v) in a borosilicate photoreactor (total volume 7.74 mL). Prior to photocatalytic testing, the reaction solution was purged for 20 min with CO_2 containing 2% methane as internal standard for gas chromatography. The vials were then irradiated using a solar light simulator with a Xe lamp (100 mW cm^{-2}, Newport Oriel) equipped with an air mass 1.5 global filter (AM 1.5G), infrared water filter, and a UV filter (λ > 400 nm) at 25 °C. The UV filter was removed to test the photocatalytic activity under full solar spectrum (λ > 300 nm). The performance of the system was optimized by varying the amount of catalyst in the reactor (Table S1 and Figure S27); 2 mg mpg-CN_x|CoPPc in 3 mL solvent was determined to be the optimal concentration as higher catalyst concentrations limits light absorption due to scattering.

The head space above the reaction solution was sampled with a gas-tight syringe at different time intervals for product analysis using a Shimadzu Tracera GC-2010 Plus gas chromatograph. A Hayesep D (2m * 1/8'' OD * 2mm ID, 80/100 mesh, Analytical Columns) precolumn was used to block CO_2 and H_2O, and a RT-Molsieve 5A (30m * 0.53 mm ID, Restek) main column was used to separate H_2, O_2, N_2, CH_4 and CO. The He (5.0, BOC) carrier gas was purified (HP2-220, VICI) prior to entering the GC. The column temperature was kept constant at 85 °C and the detector temperature at 300 °C. Figure S13 shows a representative gas chromatogram and the mass spectra of the headspace gas mixture. Mass spectrometry was performed on a Hiden Analytical HPR-20 benchtop gas analysis system fitted with a custom-designed 8-way microflow capillary inlet to a HAL 101 RC electron impact quadrupolar mass spectrometer with a Faraday detector.

After photocatalysis, the catalyst was removed by centrifugation and the supernatant was analyzed by ion chromatography (IC). Typically, 100 µL liquid was diluted to 1 mL and analyzed by Metrohm 882 Compact IC Plus ion chromatograph with a conductivity detector. Only trace amounts of $[HCO_2]^-$ were detected for all samples (<1 µmol g^{-1}).

For the isotopic labelling experiment, a suspension of 2 mg mpg-CN_x|CoPPc in MeCN/TEOA (3 mL) in a photoreactor was purged with N_2 followed by exchanging the headspace with $^{12}CO_2/^{13}CO_2$ via evacuation and refilling. After 12 h irradiation under visible light, the headspace gas was then transferred into an evacuated gas IR cell (SpecAc, 10 cm path length, equipped with KBr windows) and a high- resolution transmission FT-IR spectrum was collected on a Thermo Scientific Nicolet iS50 FT-IR spectrometer. Formation of ^{13}CO was further confirmed by the m/z = 29 peak in mass spectrum of the headspace gas mixture.

Recycling Tests. mpg-CN_x|CoPPc (2 mg) was dispersed in 3 mL MeCN/TEOA (4:1, v/v), purged with CO_2 (2% CH_4), and irradiated with visible light (λ > 400 nm). After 12 h, the catalyst was collected by centrifugation at 10,000 rpm, washed with MeCN (2×5 mL) and acetone (2×5 mL), redispersed in fresh MeCN/TEOA solution (3 mL), purged with CO_2, and irradiated again for subsequent runs.

For long-term recycling runs (10×4 h), a larger amount of mpg-CN_x|CoPPc (8 mg) was used to minimise the effect of loss of material during centrifugation and subsequent washing (Figure 4D).

External Quantum Efficiency (EQE) Measurement. The external quantum efficiency (EQE) was determined using simulated solar light simulator (LOT LSN 254) equipped with a monochromator (LOT MSH 300). Samples were irradiated at different wavelengths (λ = 360, 380 and 400 nm) at a light intensity (I) of I ~ 4.0 mW cm^{-2} (exact intensity was checked after each experiment, Table S4). EQE was calculated using the following equation:

$$\mathrm{EQE}\ (\%) = \frac{2 n_{CO} N_A h c}{t_{irr} I \lambda A} \times 100$$

where, n_{CO} is number of moles of CO produced, N_A is Avogadro's number, h is Planck's constant, c is speed of light, t_{irr} is reaction time, *I* is intensity of light, λ is the wavelength of incident light, and A is cross-sectional area of irradiation.

Supporting Figures

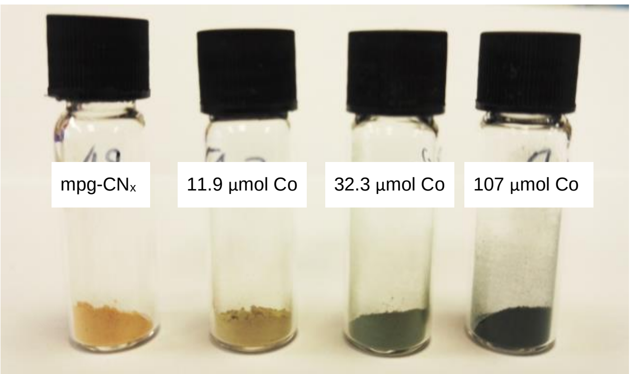

Figure S1. Photograph of mpg-CNx|CoPPc with different catalysts loadings.

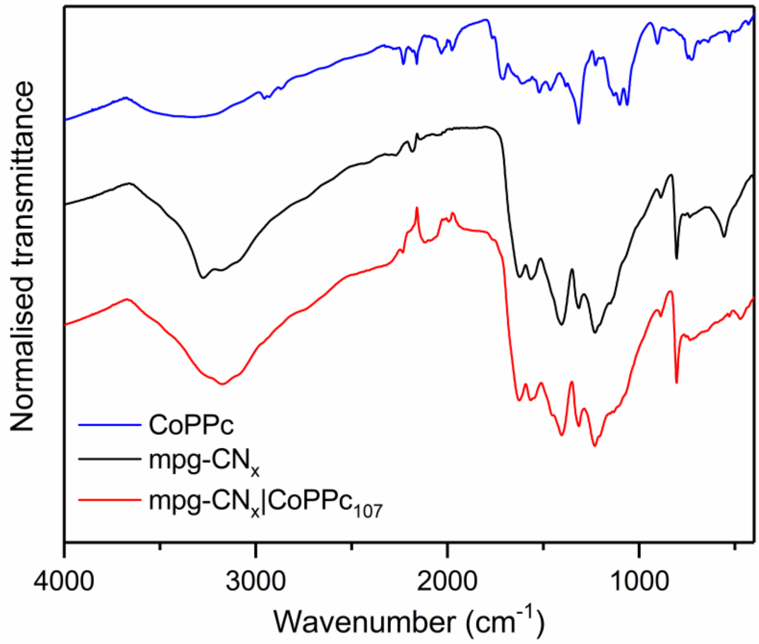

Figure S2. ATR-IR spectra of unmodified mpg-CNx, pure CoPPc, and mpg-CNx|CoPPc$_{107}$.

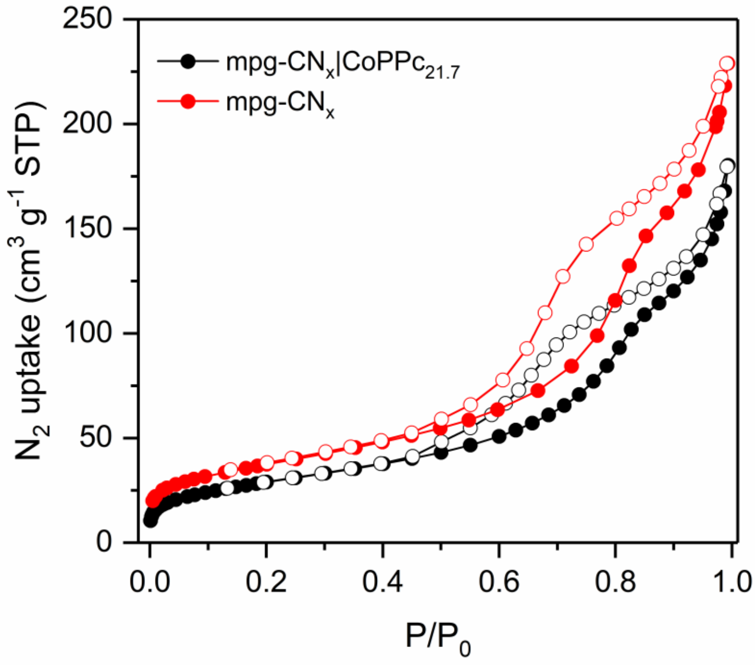

Figure S3. N_2 sorption isotherm of mpg-CN_x and mpg-CN_x|$CoPPc_{21.7}$ at 77 K. Solid and open circles denote adsorption and desorption respectively. Pore volume of mpg-CNx and mpg-CN_x|$CoPPc_{21.7}$ were calculated to be 0.35 $cm^3\ g^{-1}$ and 0.28 $cm^3\ g^{-1}$, respectively.

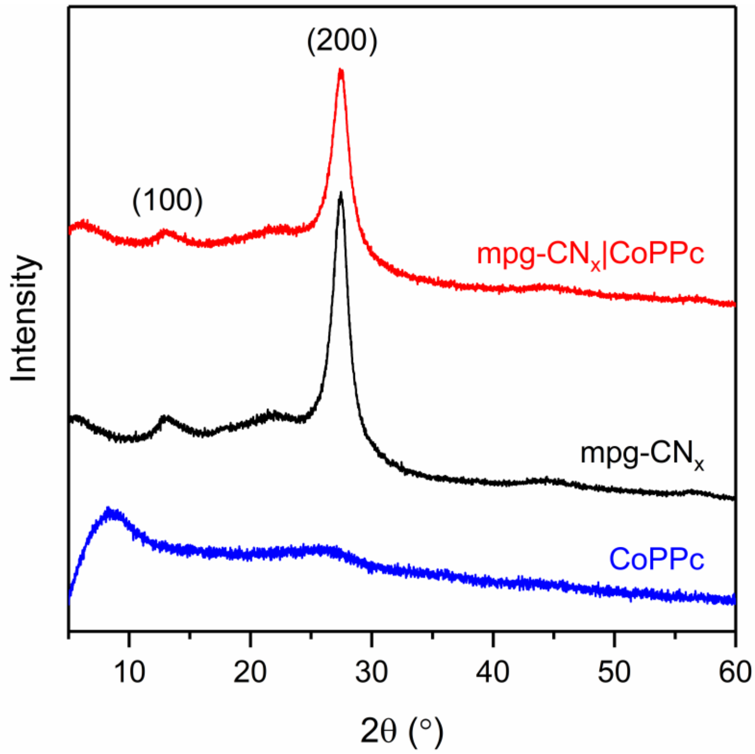

Figure S4. Powder X-ray diffraction patterns of mpg-CNx and mpg-CN_x|$CoPPc_{107}$.

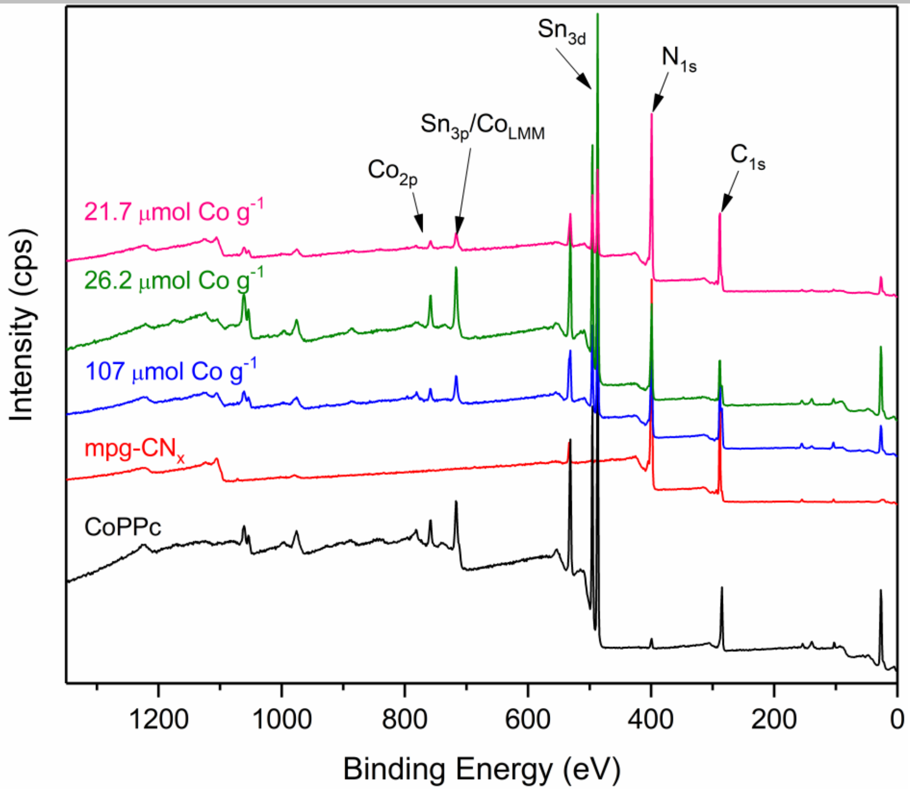

Figure S5. XPS survey spectra of mpg-CN_x, pure CoPPc polymer, and mpg-CN_x|CoPPc with different catalyst loadings.

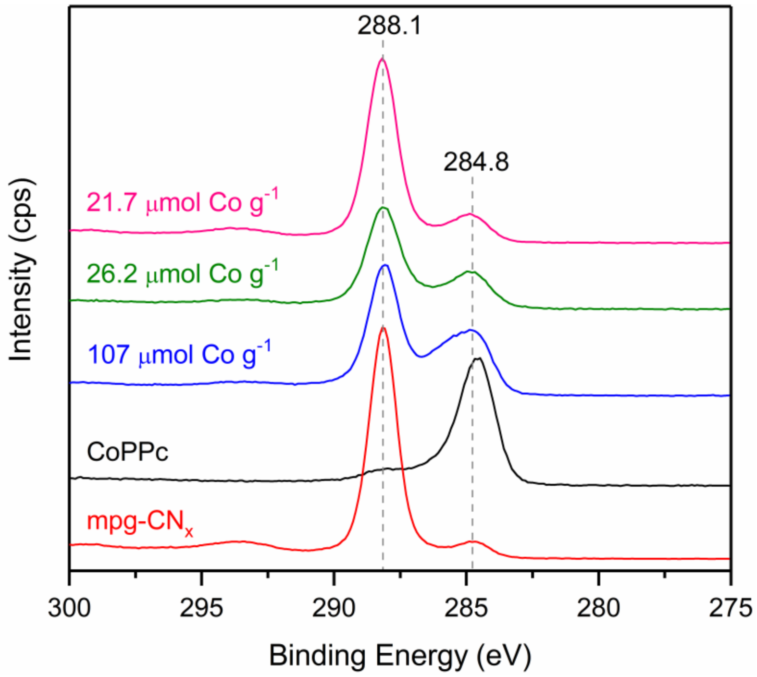

Figure S6. C1s XPS spectra of mpg-CN_x, pure CoPPc polymer, and mpg-CN_x|CoPPc with different Co loadings.

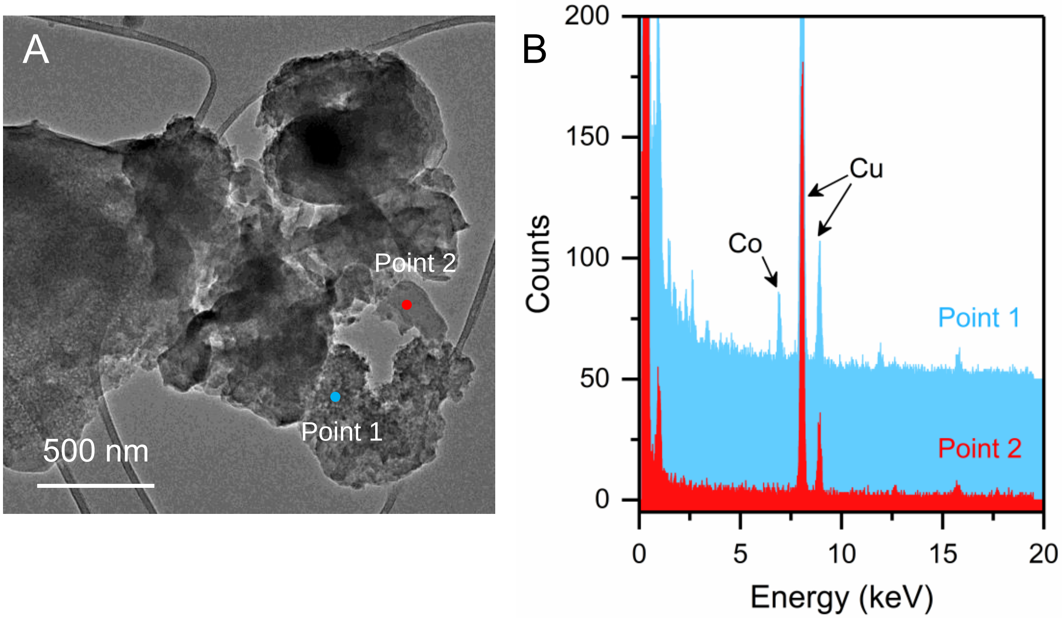

Figure S7. (A) TEM image of mpg-CN_x|$CoPPc_{17.4}$ showing the distribution of CoPPc polymer which appears as a network and confirmed by EDX point analysis. (B) Representative EDX spectra of CoPPc modified region (point 1) and pristine mpg-CN_x region (point 2). The copper peak originates from the grid used for TEM measurements.

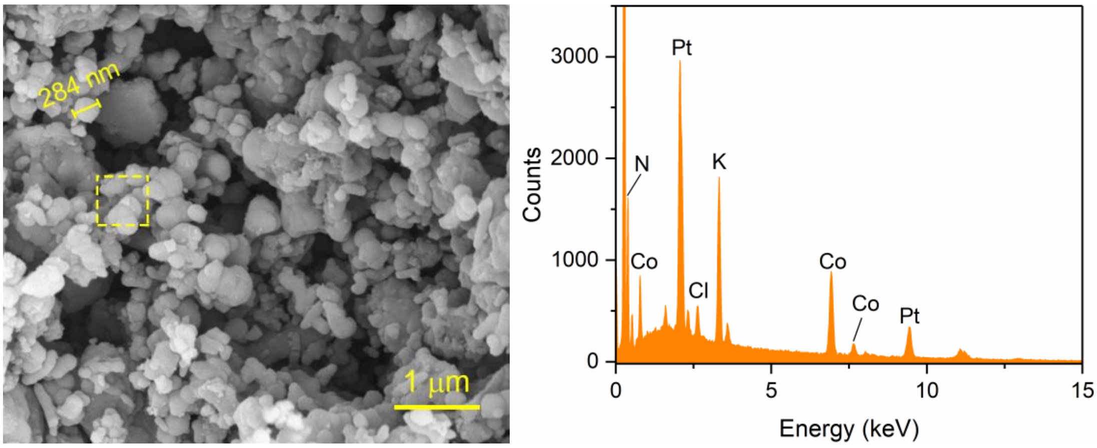

Figure S8. SEM image of mpg-CN_x|$CoPPc_{107}$ and corresponding EDS spectrum of the region shown in yellow box (Pt peaks are caused by sputtering).

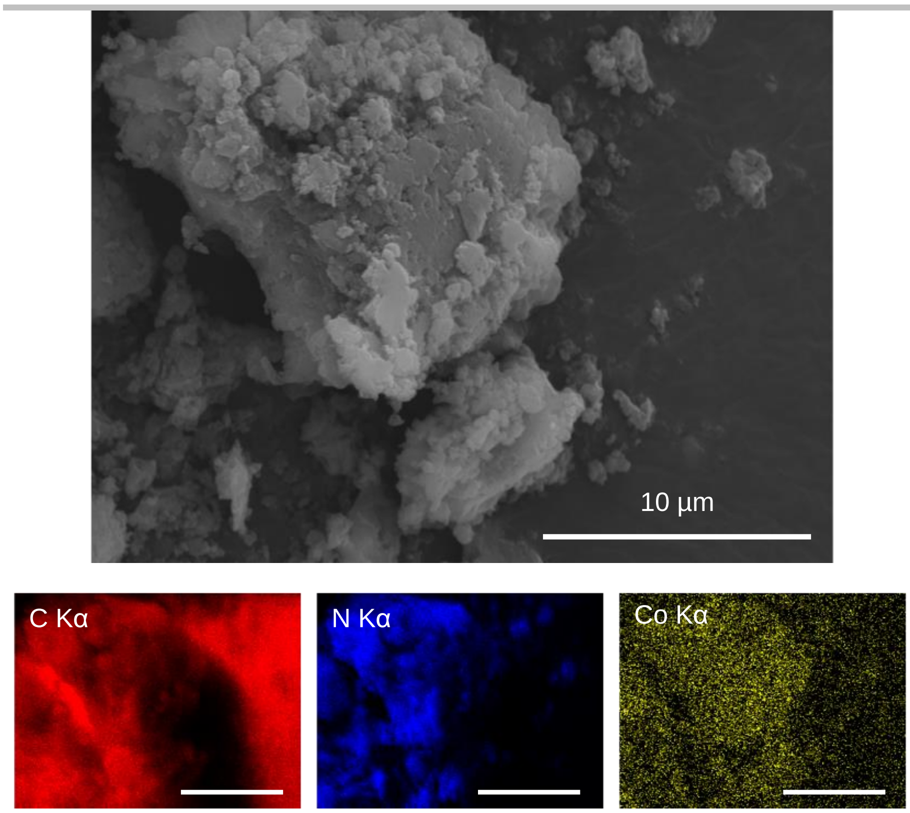

Figure S9. SEM image and corresponding C, N and Co EDS elemental mapping of mpg-CN_x|$CoPPc_{107}$ (scale bars represent 10 µm).

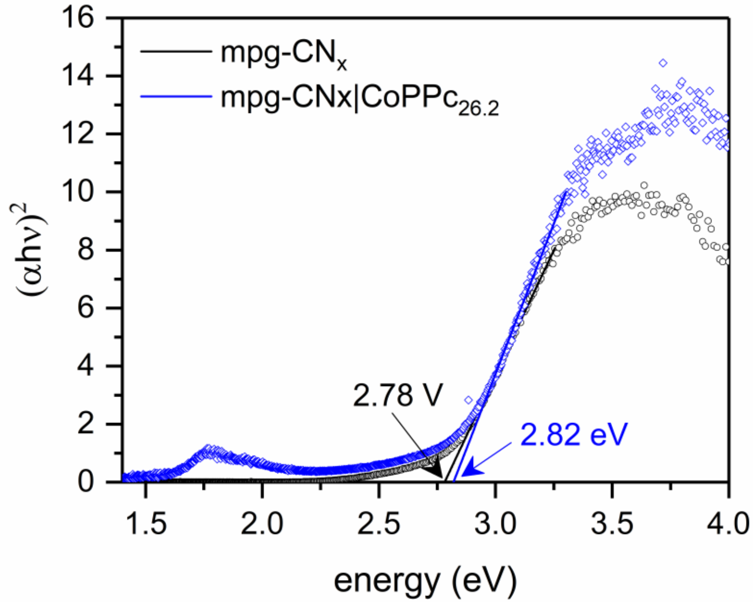

Figure S10. Bandgap estimation of mpg-CN_x and mpg-CN_x|$CoPPc_{26.2}$ from $(\alpha h\upsilon)^2$ vs photon energy plot derived from the diffuse reflectance spectra.

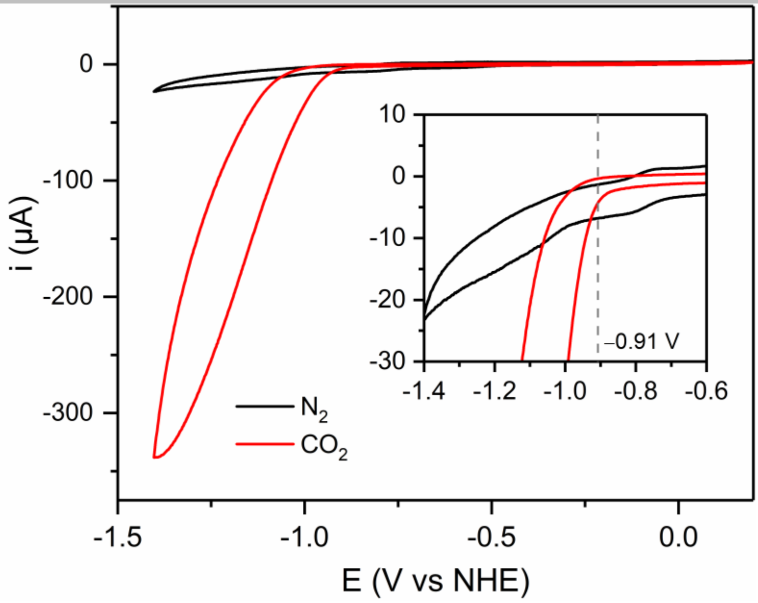

Figure S11. Cyclic voltammograms of CoPPc|CNT composite (CNT = multiwalled carbon nanotube) in 4:1 CH_3CN/TEOA solution (0.1 M NBu_4PF_6) at v= 0.1 V s^{-1} under N_2 (black) and CO_2 (red). Inset figure shows the onset potential for electrocatalytic CO_2 reduction in 4:1 CH_3CN/TEOA.

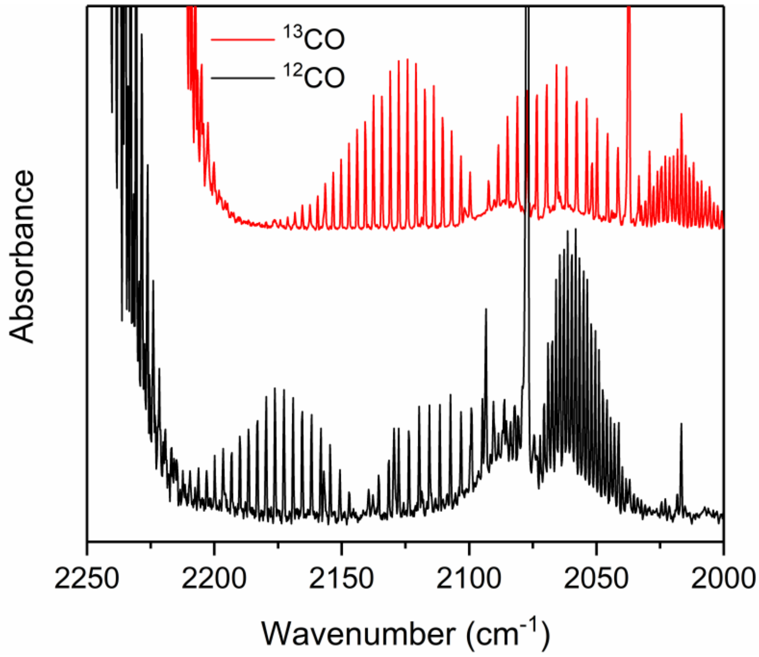

Figure S12. Isotopic labelling control experiment: IR spectra of samples of the gaseous products taken after 12 h visible light irradiation (λ > 400 nm, AM 1.5G, 100 mW cm^{-2}) under $^{12}CO_2$ and a $^{13}CO_2$ environment. Condition: 5 mg mpg-CN_x|$CoPPc_{11.9}$ suspended in 3 mL 4:1 MeCN/TEOA (v/v).

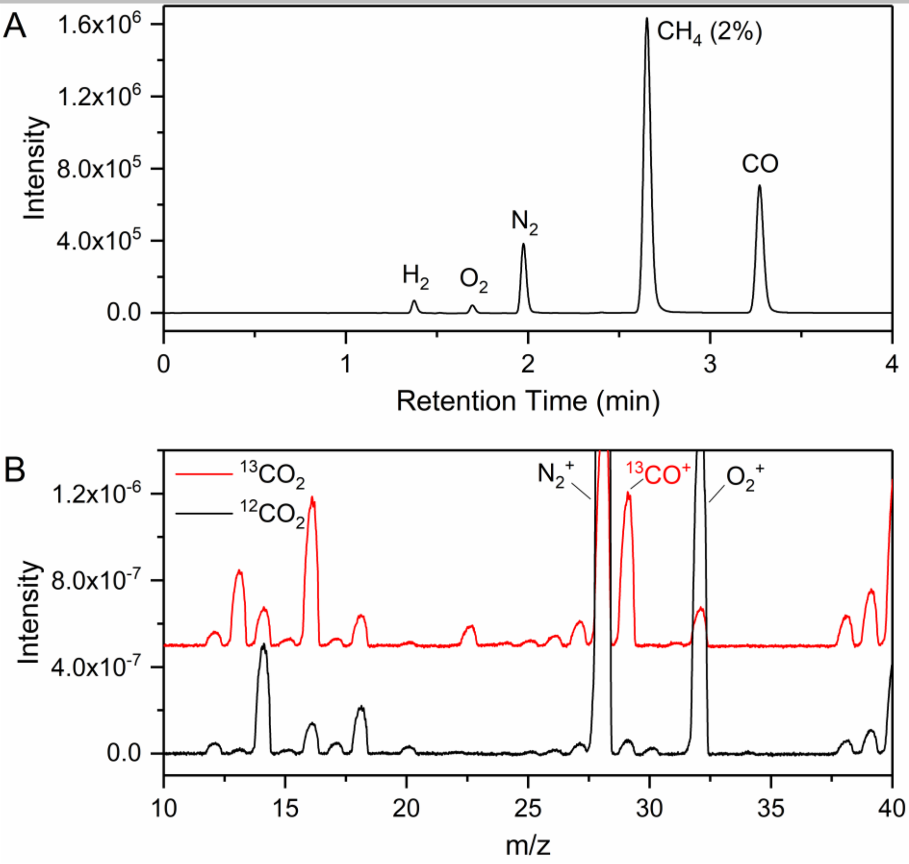

Figure S13. (A) Typical gas chromatogram observed after 24 h irradiation of a CO_2 saturated suspension of mpg-CN_x|$CoPPc_{11.9}$ in an acetonitrile/TEOA (4:1, v/v) mixture (λ > 300 nm, AM 1.5G, 100 mW cm^{-2}). (B) Mass-spectra of the headspace gas mixture recorded after 24 h irradiation under $^{12}CO_2$ and a $^{13}CO_2$ environment.

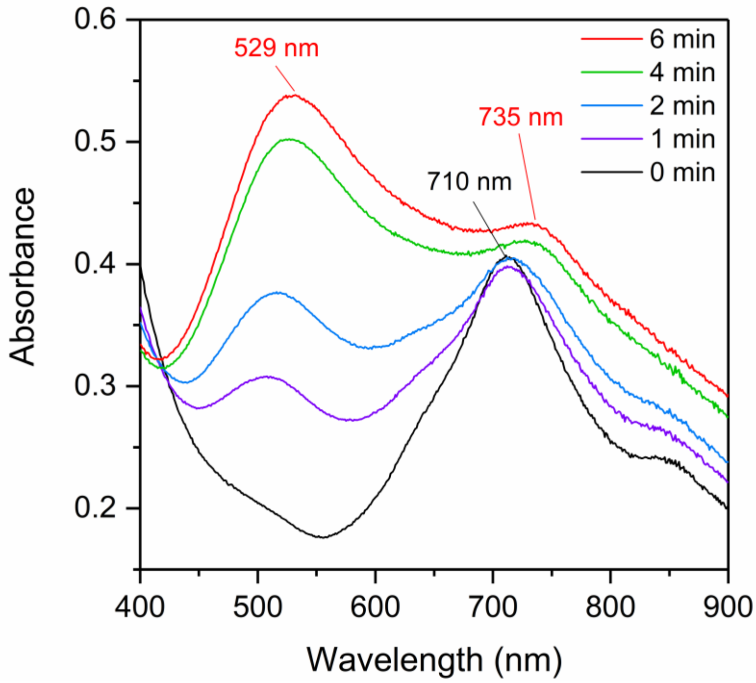

Figure S14. Transmission UV-Vis spectra of CoPPc deposited on an FTO-coated glass electrode in a N_2-saturated acetonitrile solution containing 0.1 M NBu_4^+ PF_6^- (black) and upon applying −1.0 V vs. NHE.

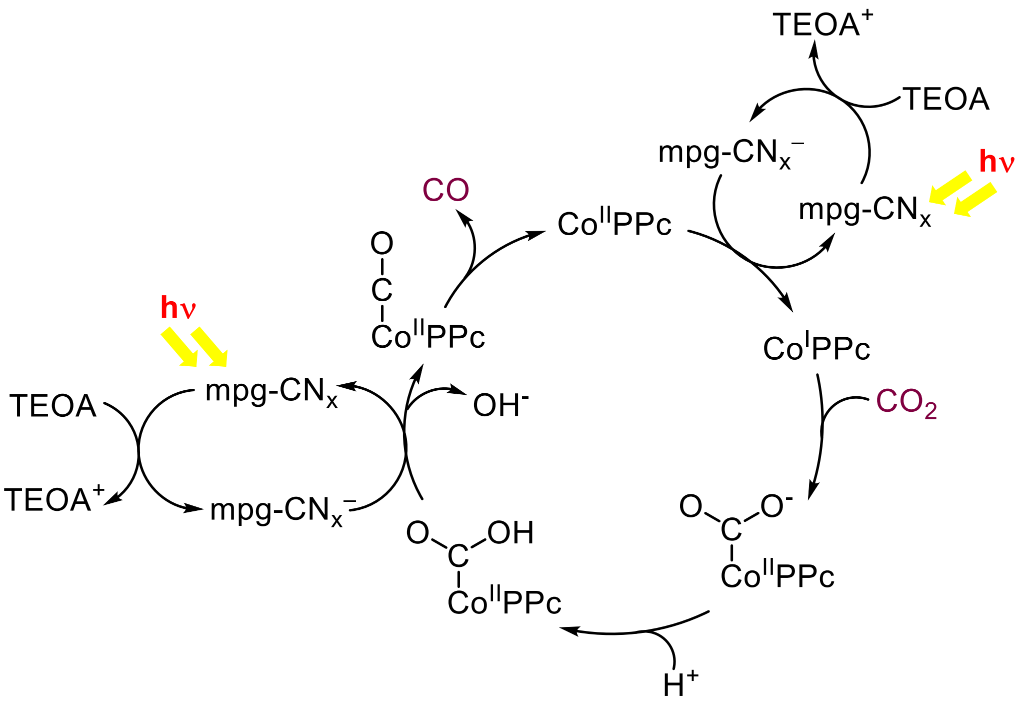

Figure S15. Proposed mechanistic scheme for CO_2 reduction by mpg-CN_x|CoPPc.[3]

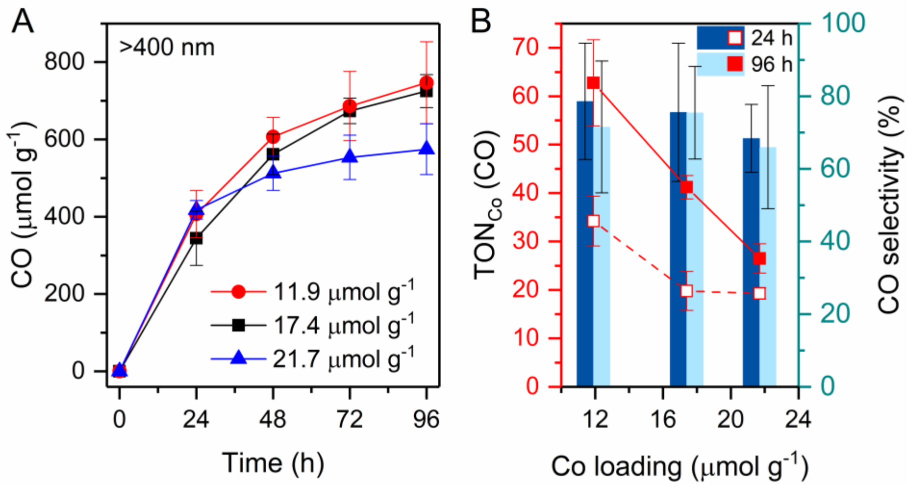

Figure S16. (A) Long-term performance (4 days) of mpg-CN_x|CoPPc with three different Co loadings under visible light irradiation (λ > 400 nm, AM 1.5G, 100 mW cm^{-2}) in MeCN/TEOA. (B) Catalytic turnover numbers (TON_{Co} (CO), red traces) and selectivity (bar plot) and for CO evolution after 24 h and 96 h. The dashed and solid red traces display TONs after 24 h and 96 h respectively.

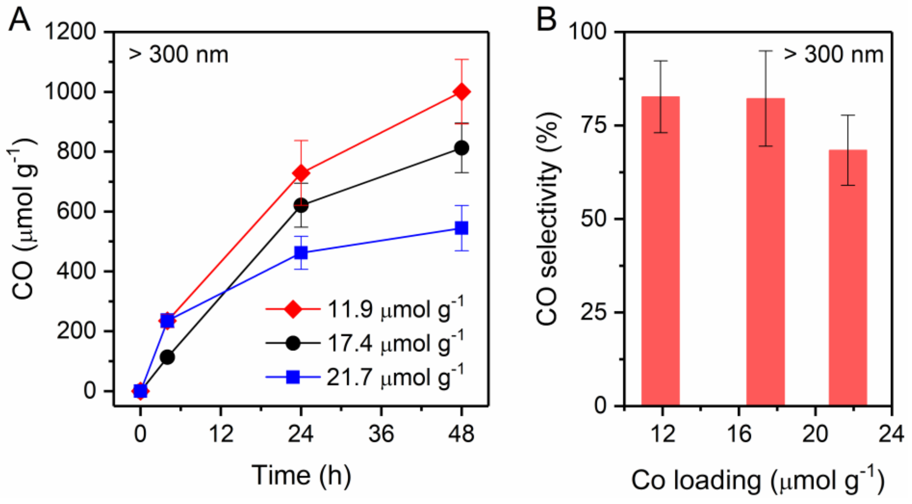

Figure S17. (A) CO production using mpg-CN_x|CoPPc with three different catalyst loadings under UV-visible light (λ > 300 nm, AM 1.5 G, 100 mW cm^{-2}). (B) Variation of selectivity towards CO with Co-loading after 24 h. Condition: ~2 mg mpg-CN_x|$CoPPc_a$ (a = 11.9, 17.4, 21.7 µmol Co g^{-1}) suspended in 3 mL 4:1 MECN/TEOA (v/v) and irradiated for 48 h.

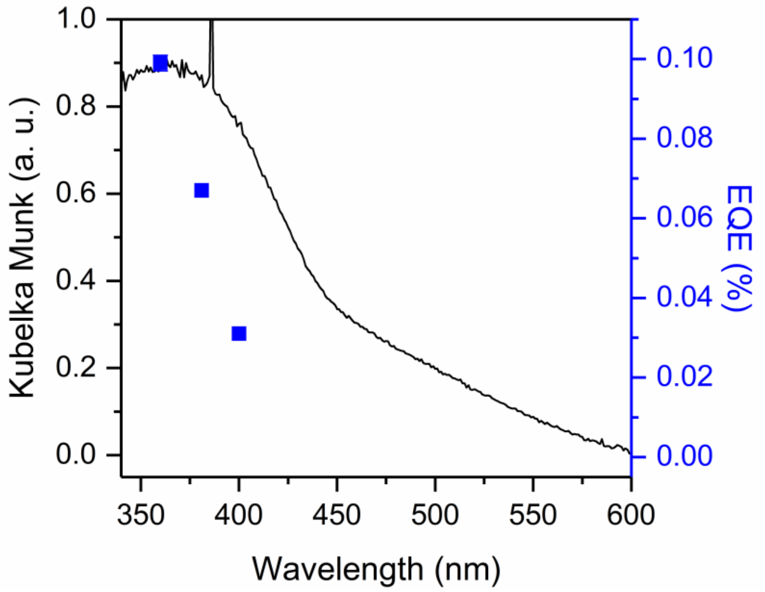

Figure S18. Diffuse reflectance UV-vis spectrum of mpg-CN_x with EQE values for mpg-CN_x|$CoPPc_{11.9}$ determined at λ = 360, 380 and 460 nm (Table S4).

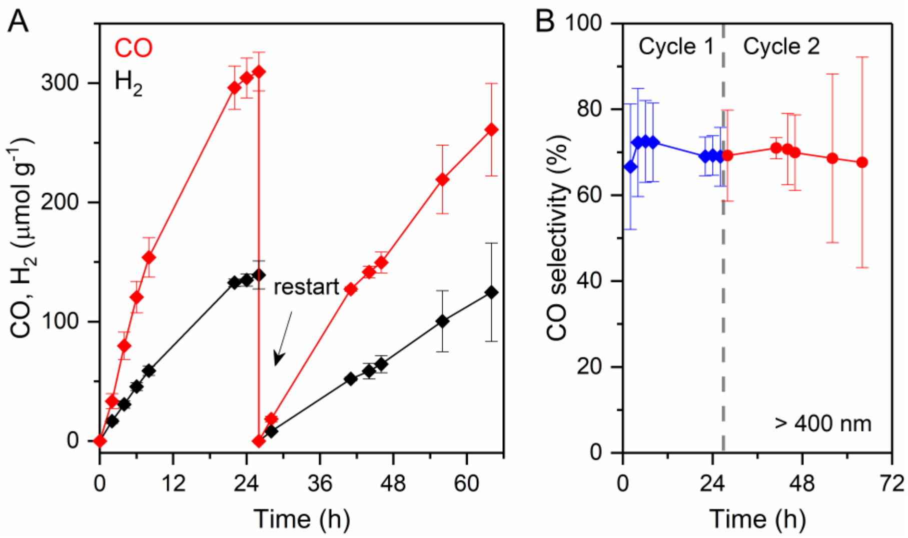

Figure S19. (A) CO production reduction for two cycles (cycle 1: 26 h, cycle 2: 38 h) using 6 mg mpg-CN_x|$CoPPc_{21.7}$ in 3.0 mL acetonitrile containing TEOA (λ > 400 nm, AM 1.5G 100 mW cm^{-2}). (B) Selectivity for CO production over two cycles. After photocatalysis for 26 h, the photocatalyst was collected by centrifugation, washed with acetonitrile, and re-dispersed in 3.0 mL fresh acetonitrile containing TEOA. Photocatalysis was then repeated under the same conditions after purging with CO_2.

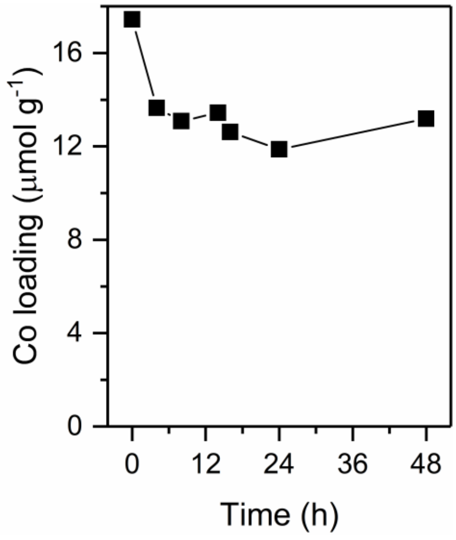

Figure S20. Cobalt loss from mpg-CN_x|$CoPPc_{17.6}$ during 48 h photocatalysis under visible light (λ > 400 nm, AM 1.5G 100 mW cm^{-2}). The solid catalysts were isolated after running photocatalysis for a specific duration, followed by acid digestion and ICP-OES analysis. Condition: six reactors containing ~2 mg mpg-CN_x|$CoPPc_{11.9}$ in 3 mL 4:1 MeCN/TEOA (v/v) were irradiated for 4, 8, 14, 16, 24, and 48 h.

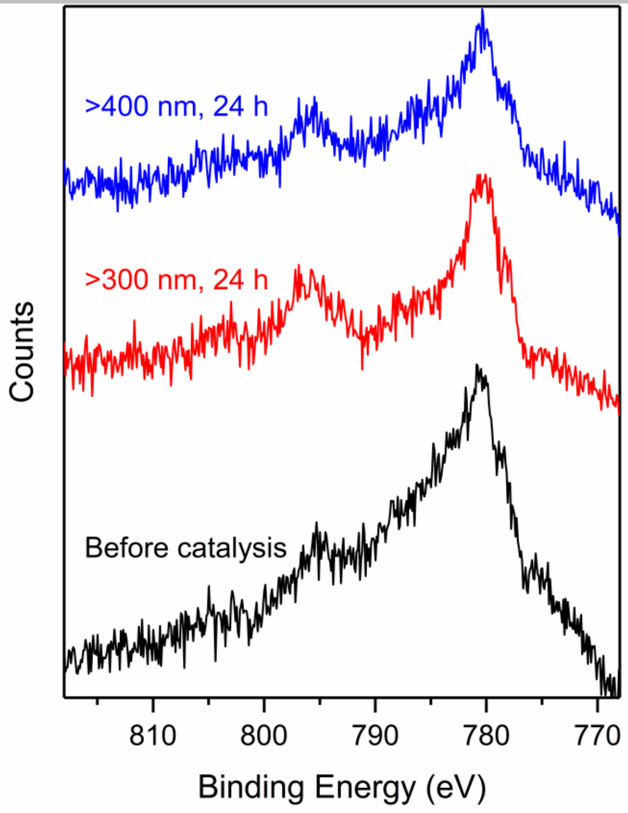

Figure S21. Co 2p core level XPS spectra of mpg-CN_x|$CoPPc_{17.4}$ before and after 24 h photocatalysis under visible (λ > 400 nm, AM 1.5G, 100 mW cm^{-2}, blue trace) and UV-visible (λ > 300 nm, AM 1.5G, 100 mW cm^{-2}, red trace) light irradiation in CO_2 saturated MeCN/TEOA (4:1 v/v). Condition: ~3 mg catalyst was suspended in 3 mL 4:1 MeCN/TEOA (v/v) and irradiated for 24 h.

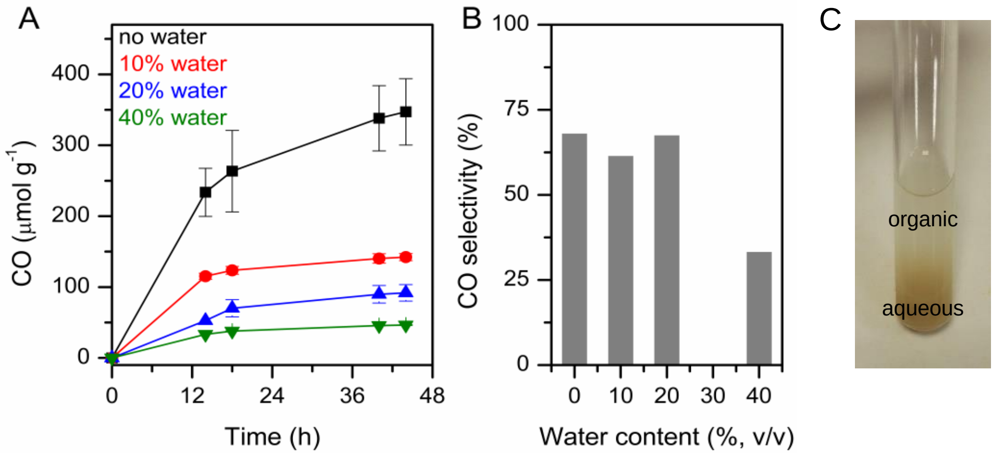

Figure S22. (A) CO production by mpg-CN_x|$CoPPc_{22}$ in aqueous acetonitrile in the presence of TEOA (20% v/v) under visible light (λ > 400 nm, AM 1.5G, 100 mW cm^{-2}). (B) Selectivity towards CO formation in the presence of water. (C) Photograph of the photocatalysis solution containing 20% water after 18 h irradiation. Condition: ~2 mg mpg-CN_x|$CoPPc_{21.7}$ suspended in 3 mL MeCN/water/TEOA mixture and irradiated for 44 h.

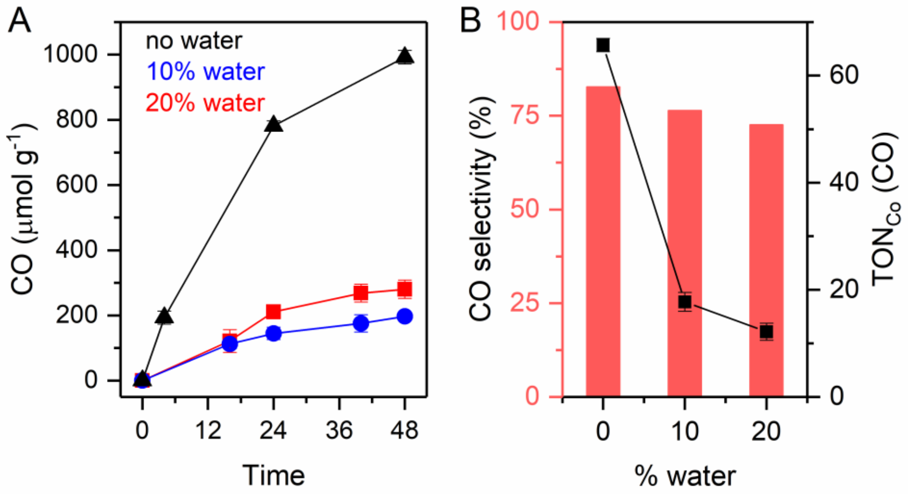

Figure S23. (A) CO production by mpg-CN_x|$CoPPc_{11.9}$ in aqueous MeCN in the presence of TEOA (20% v/v) under UV-visible light (λ > 300 nm, AM 1.5G, 100 mW cm^{-2}). (B) Selectivity towards CO formation (bar plot) and TON (CO) per cobalt (black trace) in the presence of water. Condition: ~2 mg mpg-CN_x|$CoPPc_{11.9}$ suspended in 3 mL MeCN/water/TEOA (20% v/v) mixture and irradiated for 48 h.

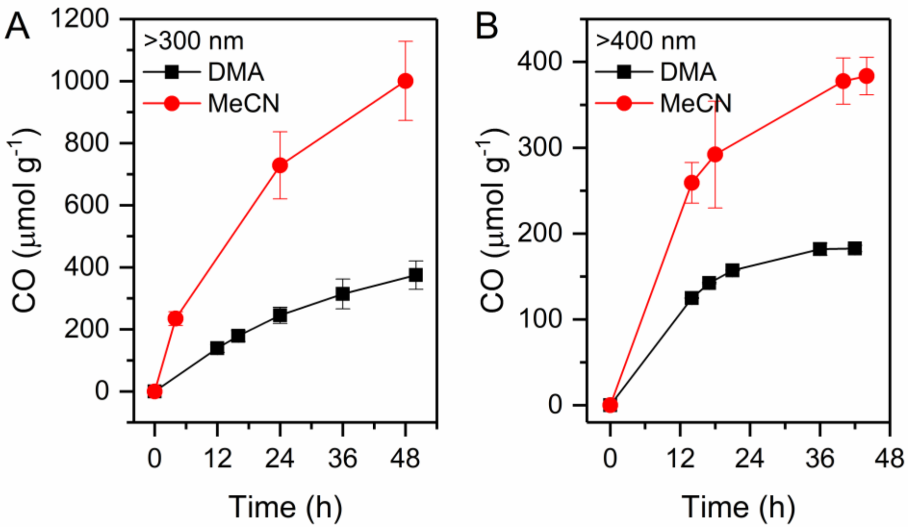

Figure S24. Comparison of photocatalytic activity of mpg-CN_x|$CoPPc_{11.9}$ in neat acetonitrile (MeCN) and dimethylacetamide (DMA) under (A) UV-visible (λ > 300 nm, AM 1.5G, 100 mW cm^{-2}) and (B) visible light irradiation (λ > 400 nm, AM 1.5G, 100 mW cm^{-2}). Condition: ~2 mg mpg-CN_x|$CoPPc_{11.9}$ suspended in 3 mL 4:1 MeCN/TEOA or 4:1 DMA/TEOA (v/v) and irradiated for 48 h.

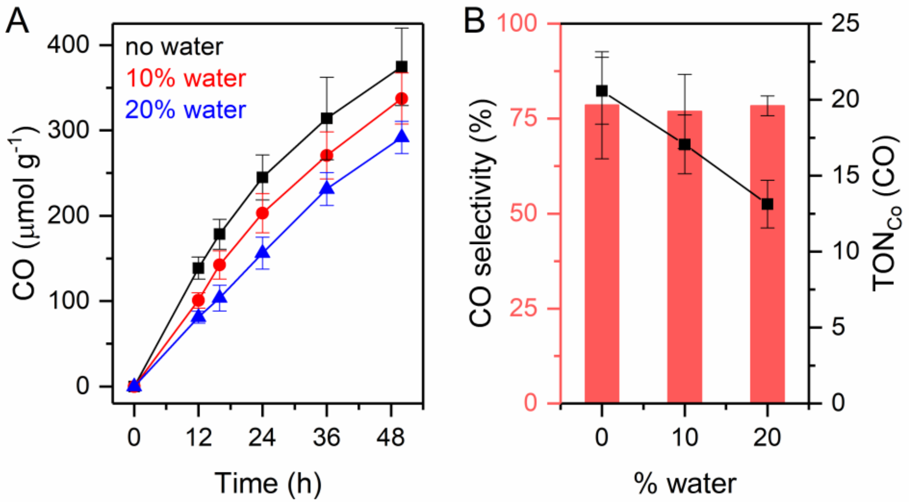

Figure S25. (A) CO production by mpg-CN_x|$CoPPc_{11.9}$ in aqueous dimethylacetamide (DMA) in the presence of TEOA (20% v/v) under UV-visible light (λ > 300 nm, AM 1.5G, 100 mW cm^{-2}). (B) Selectivity towards CO formation (bar plot) and TON_{Co} (CO) (black trace) in the presence of water. Condition: ~2 mg mpg-CN_x|$CoPPc_{11.9}$ suspended in 3 mL DMA/water/TEOA (20% v/v) mixture and irradiated for 50 h.

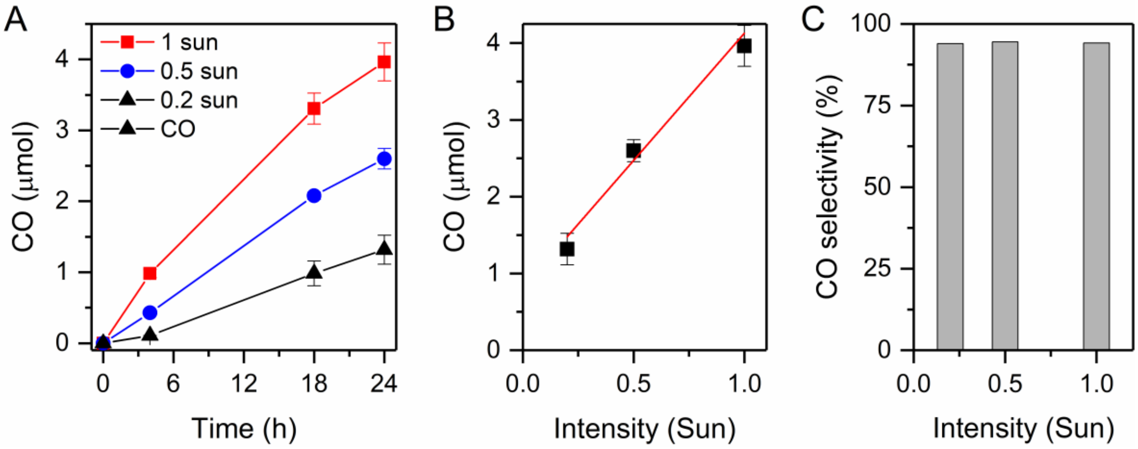

Figure S26. (A) Dependence of rate of CO evolution on the light intensity. Fig. (B) shows the linear correlation between the amount of CO evolved after 24 h and the light intensity. Fig. (C) shows that the selectivity remained constant. Condition: 5 mg mpg-CN_x|$CoPPc_{22}$ suspended in 3 mL MeCN/TEOA (4:1, v/v) and irradiated under UV-visible light (λ > 300 nm, AM 1.5G, 100 mW cm^{-2}) using appropriate neutral density filters (0.5 and 0.8 OD for 0.5 and 0.2 Sun, respectively; no filter was used for 1 sun irradiation).

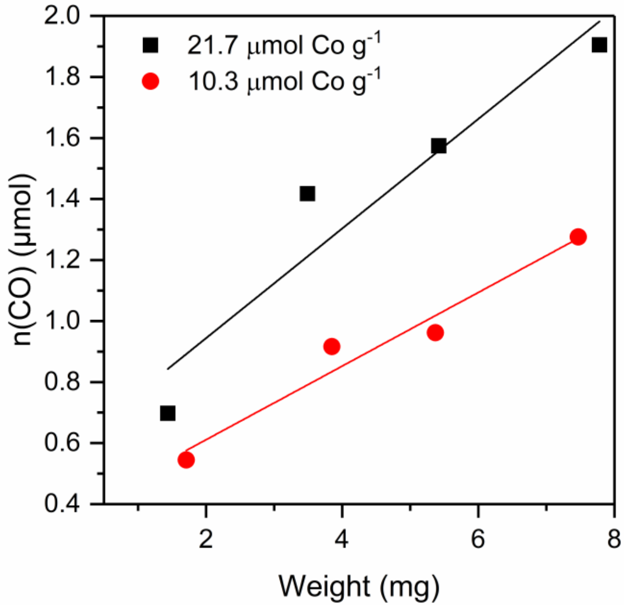

Figure S27. Dependence of CO evolved on the amount of mpg-CN_x|CoPPc used in the photoreactor. Catalyst was suspended in 3 mL MeCN/TEOA and irradiated by visible light (λ>400 nm cut off filter, AM 1.5G, 100 mW cm^{-2}) for 24 h

Table S1. Optimization of CO_2 reduction activity of mpg-CN_x|CoPPc with respect to the weight of catalyst used in the reactor. Catalyst was suspended in 3 ml MeCN/TEOA and irradiated by visible light (λ>400 nm cut off filter, AM 1.5G, 100 mW cm^{-2}) for 24 h.

Co loading (µmol g^{-1})	Weight (mg)	n(CO) (µmol)	CO (µmol g^{-1})
21.7	1.44	0.6978	484.58
	3.49	1.4180	406.30
	5.42	1.5741	290.42
	7.78	1.9053	244.90
10.3	1.71	0.5449	318.65
	3.85	0.9167	238.10
	5.37	0.9620	179.14
	7.47	1.2763	170.86

Table S2. CO_2 reduction mpg-CN_x|CoPPc with varying catalyst loading in MeCN/TEOA (4:1, v/v) under 1 sun irradiation (100 mW cm^{-2}) equipped with an UV-filter (λ > 400 nm). Error bars are calculated based on triplicate measurements.

Entry	Photosensitizer	Co loading (µmol g^{-1})	Wavelength	Irradiation time (h)	CO (µmol g^{-1})	TON_{Co} (CO)	H_2 (µmol g^{-1})	CO selectivity (% yield)
1	mpg-CN_x	0	>400 nm	24	3.8±2.1	-	7.2±2.3	34.4±22.2
2	g-CN_x	20	>400 nm	24	11.4±2.8	0.6±0.1	55.7±9.9	17.0±6.2
3	mpg-CN_x	4.1	>400 nm	24	104.0±28.7	25.4±6.0	189.3±36.4	35.5±13.1
4	mpg-CN_x	7.1	>400 nm	24	184.8±36.0	26.0±4.5	110.7±7.6	62.5±12.9
5	mpg-CN_x	10.3	>400 nm	24	173.3±23.4	16.8±2.0	70.7±14.3	71.0±18.9
6	mpg-CN_x	11.9	>400 nm	24	455.8±33.5	38.5±2.8	111.9±13.4	80.3±11.3
7	mpg-CN_x	17.4	>400 nm	24	345.0±48.0	19.8±2.8	111.3±16.5	75.6±15.4
8	mpg-CN_x	21.7	>400 nm	24	339.5±31.7	15.6±1.4	136.9±21.2	71.3±10.6
9	mpg-CN_x	26.2	>400 nm	24	153.7±15.02	5.9±0.6	104.9±4.0	59.4±6.2
10	mpg-CN_x	32.3	>400 nm	24	205.2±34.22	6.3±1.7	285.7±44.6	41.8±12.8
11	mpg-CN_x	107	>400 nm	24	5.8±7.91	<0.1	63.9±3.6	8.4±11.4
12	mpg-CN_x	11.9	>400 nm	96	746.8±105.8	62.8.7±8.9	297.3±62.6	72.5±17.3
13	mpg-CN_x	17.4	>400 nm	96	725.1±42.7	41.7±2.4	236.3±37.4	75.4±12.7
14	mpg-CN_x	21.7	>400 nm	96	574.7±65.6	26.5±3.0	296.7±68.2	65.7±16.8

Table S3. Comparison of CO_2 reduction activity by mpg-CN_x|CoPPc with three different Co loadings under visible (λ>400 nm) and UV-visible light (λ>300 nm) with an intensity of 100 mW cm^{-2}. Error bars are calculated based on triplicate measurements.

Entry	Material	Co loading (μmol g^{-1})	Wavelength	Irradiation time (h)	CO (μmol g^{-1})	TON_{Co} (CO)	H_2 (μmol g^{-1})	CO selectivity (% yield)
1	mpg-CN_x	11.9	>300 nm	48	1000.6±107.6	84.1±9.0	181.8±16.9	84.7±14.0
2			>300 nm	60	1076.2±193.2	90.4±16.2	193.4±30.3	84.8±20.2
3			>400 nm	48	606.7±50.0	51.0±4.2	192.5±30.2	75.9±12.5
4			>455 nm	24	10.4±2.4	0.8±0.2	10.3±1.4	50.2±18.3
5	mpg-CN_x	17.4	>300 nm	48	813.0±82.7	46.7±4.7	201.3±13.3	80.1±9.7
6			>400 nm	48	561.6±52.3	31.9±3.0	171.1±5.7	76.6±7.6
7	mpg-CN_x	21.7	>300 nm	48	544.9±75.4	25.1±3.5	320.8±25.1	67.4±10.7
8			>400 nm	48	512.4±44.4	23.6±2.0	270.2±23.7	65.5±8.1

Table S4. Estimated quantum efficiency (EQE) of CO production under various reaction conditions in MeCN/TEOA mixture (reaction volume 1 mL

Entry	Catalyst	Weight (mg)	Wavelength (nm)	Intensity (mW cm^{-2})	Time (h)	CO (μmol)	EQE (%)
1	mpg-CN_x|$CoPPc_{11.9}$	1.9	360	3.8	4	0.09	0.107
2	mpg-CN_x|$CoPPc_{11.9}$	2.1	360	3.8	12	0.25	0.101
3	mpg-CN_x|$CoPPc_{11.9}$	2.0	380	3.5	4	0.06	0.072
4	mpg-CN_x|$CoPPc_{11.9}$	1.9	400	3.6	12	0.09	0.034

Table S5. Quantification of cobalt in mpg-CN_x|CoPPc by ICP-OES

Sample	Weight [a]	[Co],[a] ppm	Co, wt.%	Co μmol g^{-1}
mpg-CN_x|$CoPPc_{4.1}$	2.46	0.058	0.0241	4.09
	2.72	0.067		
mpg-CN_x|$CoPPc_{7.1}$	2.53	0.105	0.0419	7.10
	2.14	0.090		
mpg-CN_x|$CoPPc_{10.3}$	3.48	0.212	0.0607	10.29
	3.22	0.195		
mpg-CN_x|$CoPPc_{11.9}$	1.98	0.138	0.0703	11.92
	1.77	0.126		
mpg-CN_x|$CoPPc_{17.4}$	3.50	0.360	0.1028	17.42
	2.60	0.267		
mpg-CN_x|$CoPPc_{21.7}$	2.20	0.282	0.1283	21.75
	1.80	0.231		
mpg-CN_x|$CoPPc_{26.2}$	1.86	0.292	0.1546	26.18
	2.10	0.319		
mpg-CN_x|$CoPPc_{32.3}$	1.22	0.242	0.1908	32.33
	1.31	0.240		
mpg-CN_x|$CoPPc_{107}$	1.86	1.185	0.6333	107.35
	1.51	0.951		
CoPPc	1.36	10.494	7.4737	1266.73
	0.99	7.159		
mpg-CN_x|$CoCl_2$	3.94	0.145	0.036	5.98

[a] solid samples were digested in 2:1 mixture of 30% H_2O_2/conc. H_2SO_4 and diluted to a total volume of 10 mL.

Supporting References

[1] F. Goettmann, A. Fischer, M. Antonietti, A. Thomas, *Angew. Chem. Int. Ed.* **2006**, *45*, 4467-4471.

[2] N. Han, Y. Wang, L. Ma, J. Wen, J. Li, H. Zheng, K. Nie, X. Wang, F. Zhao, Y. Li, J. Fan, J. Zhong, T. Wu, D. J. Miller, J. Lu, S.-T. Lee, Y. Li, *Chem* **2017**, *3*, 652-664.

[3] M. Zhu, R. Ye, K. Jin, N. Lazouski, K. Manthiram, *ACS Energy Lett.* **2018**, *3*, 1381-1386.

End of Supporting Information
